# Depressive Emotion Detection and Behavior Analysis of Men Who Have Sex With Men *via* Social Media

**DOI:** 10.3389/fpsyt.2020.00830

**Published:** 2020-08-14

**Authors:** Yong Li, Mengsi Cai, Shuo Qin, Xin Lu

**Affiliations:** ^1^College of Economy and Management, Changsha University, Changsha, China; ^2^College of Systems Engineering, National University of Defense Technology, Changsha, China; ^3^Julius Center for Health Sciences and Primary Care, University Medical Center Utrecht, Utrecht, Netherlands; ^4^National Key Laboratory of Science and Technology on Blind Signal Processing, Chengdu, China; ^5^School of Business, Central South University, Changsha, China

**Keywords:** depressive emotion detection, men who have sex with men, behavior analysis, Blued, Twitter

## Abstract

**Background:**

A large amount of evidence has indicated an association between depression and HIV risk among men who have sex with men (MSM), but traditional questionnaire-based methods are limited in timely monitoring depressive emotions with large sample sizes. With the development of social media and machine learning techniques, MSM depression can be well monitored in an online and easy-to-use manner. Thereby, we adopt a machine learning algorithm for MSM depressive emotion detection and behavior analysis with online social networking data.

**Methods:**

A large-scale MSM data set including 664,335 users and over 12 million posts was collected from the most popular MSM-oriented geosocial networking mobile application named Blued. Also, a non-MSM Benchmark data set from Twitter was used. After data preprocessing and feature extraction of these two data sets, a machine learning algorithm named XGBoost was adopted for detecting depressive emotions.

**Results:**

The algorithm shows good performance in the Blued and Twitter data sets. And three extracted features significantly affecting the depressive emotion detection were found, including depressive words, LDA topic words, and post-time distribution. On the one hand, the MSM with depressive emotions published posts with more depressive words, negative words and positive words than the MSM without depressive emotions. On the other hand, in comparison with the non-MSM with depressive emotions, the MSM with depressive emotions showed more significant depressive symptoms, such as insomnia, depressive mood, and suicidal thoughts.

**Conclusions:**

The online MSM depressive emotion detection using machine learning can provide a proper and easy-to-use way in real-world applications, which help identify high-risk individuals at the early stage of depression for further diagnosis.

## Introduction

Depression is a prevalent but potentially treatable health problem and is a leading cause of disability worldwide, with more than 264 million people affected (1). Depressed people have various symptoms of depression manifested by distinguishing behaviors, such as persistent sadness, loss of interest, changes in appetite, low concentration, sleep problems, feelings of guilt or hopelessness, decreased energy, suicidal thoughts, etc. This emotional disturbance not only affects daily functions but also increases the global burden.

Men who have sex with men (MSM) are disproportionately affected by and infected with HIV/AIDS (2). Suffering from disproportionate rates of stigma and discrimination (3, 4), MSM experience remarkably poorer mental health (5). And evidence has demonstrated that HIV risk (6–9), amphetamine-type-stimulants (ATS) use and homosexuality-related stigma (10), and sexual risk behaviors (11, 12) are associated with depression among MSM population. Therefore, it is of crucial importance to develop reliable and efficient methods for detection and early warning of the depressive emotions or mental health among MSM population, and enable those suffering from depression to be more proactive about their mental health.

Current scales to assess depression have included: *Beck’s Depression Inventory (BDI)* (13) with 21 questions about users’ mental and physiological states; *Beck Depression Inventory Fast-Screen (BDI-FS)* (14), *CES-D Scale* (15) with 20 questions about mental conditions such as users’ feelings of guilt and sleep conditions; *the Patient Health Questionnaire-9 (PHQ-9)* (16) with nine questions about depressive symptoms, such as little interest and suicidal thoughts. In addition, the *Diagnostic and Statistical Manual of Mental Disorders (DSM)* (17) provides standard criterion for diagnosing depression and describes nine kinds of depressive indicators, such as depressed mood and diminished interest, etc. Although these methods have achieved effectiveness for depression diagnosis, the aforementioned criteria may not comprehensively cover newly emerging depressive behaviors and symptoms as the symptoms of depressive disorders evolve over time. n addition, people are somehow ashamed or unaware of depression such that more than 70% of people in the early stage of depression do not consult psychological doctors, and their conditions were deteriorated (18).

On the other hand, social media platforms have become an indispensable part of everyday life for MSM and others. Studies have revealed that it is possible to monitor mental health and predict depression *via* social media (19, 20). Automated analysis of social media potentially provides methods for early detection of depression (21). As a preliminary research, Park et al. (22) explored the use of language in describing depressive moods using real-time moods captured from Twitter users; they then explored the depressive behaviors of 14 active Twitter users through face-to-face interviews (23). Predictive models, which use features or variables that have been extracted from social media data, have achieved many successes in identifying depression *via* social media. For example, commonly extracted and used features include user profile, linguistic signals, sentiment, social network, social interaction, domain-specific features, time of posts, and so on (24–27). Features are then treated as independent variables in an algorithm, such as support vector machine (SVM) (28, 29), naive Bayes methods (30), random forest (31), multi-model depressive dictionary learning model (MDDL) (18), etc. Deep learning models, which have recently achieved outstanding results in many classification tasks due to their ability to learn complex non-linear functions, have also been applied in detecting depression by feeding n-gram features or word embedding of social media data as model input (32, 33). However, deep learning models generally lack interpretability and transparency in the feature extraction and decision processes, limiting their value in clinical diagnosis (34, 35). In addition to using constituent learning algorithms alone, ensemble methods, which can obtain better and consistent predictive performance, have attracted much attention in the community. For example, the Inverse Boosting Pruning Trees (IBPT), based on the AdaBoost algorithm, demonstrated a strong depression classification ability among Twitter users (25).

With massive user-generated content, social media provide a means for capturing depressive behavioral attributes relevant to an individual’s thinking, mood, communication, activities and socialization (36). The emotion and language used in social media postings may indicate feelings of worthlessness, guilt, helplessness, and self-hatred that characterize depression or high risk of depression (29). The analysis of social networking data, thus, potentially provides methods for early detection of depression (21). Therefore, in this study, we aim to predict multidimensional depressive emotions among MSM through social media with machine learning methods, which can achieve early detection of depressive emotions, and then complement and extend traditional approaches to diagnose depression. To predict depressive emotions for depression detection, we collect massive social networking data from the most popular MSM-oriented geosocial networking mobile application named Blued, and adopt a decision tree-based ensemble machine learning algorithm named XGBoost, which has been widely applied in various classification tasks with outstanding performance and can provide explainable results. In addition, we comprehensively evaluate the classification capability of XGBoost algorithm on a publicly accessible Twitter data set (18), and compare the differences in online behaviors between MSM with depressive emotions (depressed MSM) and MSM without depressive emotions (non-depressed MSM) and the differences between depressed MSM and non-MSM with depressive emotions (depressed non-MSM).

## Materials and Methods

### Data Sources

Regarding MSM and non-MSM populations, we constructed two main databases: Blued database and Twitter database, representing MSM users and non-MSM users, respectively. Blued is the largest gay social networking application in the world with over 40 million registered users. Twitter is a popular social networking platform with over 300 million active users per month and 500 million tweets per day. Blued is mainly used for seeking partners and dating, and Twitter is mainly used for social purposes and information consumption.

To predict depressive emotions (depressed or non-depressed), we need to get a batch of labeled depression and non-depression data sets to train the classification model. Also, to expand the size of depressed users, we construct an unlabeled data set named depression-candidate data set for depressive emotion detection and for behavior analysis. For each user, we obtain the profile information and an anchor post to infer the mental state. An anchor post is a post that can help identify whether or not a user has severe depressive emotions which indicate depression. Considering that each user might have published a number of posts related to depression identification, we select only one post as the final anchor post to construct our data sets. For depressed users, we select their first anchor post to ensure timely depressive emotion detection; and for depression candidates, we select their last anchor post to cover more posts for depressive emotion detection. According to clinical experience, an observation period is required for depression diagnosis; therefore, users’ posts published within one or two months before the anchor post were also obtained. Finally, our data sets contain the users’ profile information, an anchor post for each user, and all the other posts published within one or two months before the anchor post.

#### Blued Database

Targeting the MSM community, we designed elaborate crawlers *via* Scrapy, a fast web-crawling framework, to collect public social networking records from Blued. We collected about 13 million posts on Blued, concerning 664,335 users (a sub sample from Blued). These posts were published from 1st January 2012 to 31st March 2019 and saved in a local MongoDB database. To make depressive emotion detection, the Blued database was divided into three parts:

(1) Depression Data Set B_D1: Users were labeled as depressed if their anchor posts indicated that they were diagnosed with depression, and they had suicidal thoughts, e.g. “I have depression; I want to suicide!” Posts published within two months before the anchor post of the users were included in this data set.

(2) Non-Depression Data Set B_D2: Users were labeled as non-depressed if they had never posted any posts containing the character string “depress.” As the Blued data set collected in this paper contains 664,335 active users and about 13 million posts, we select the posts published from 1st February 2019 to 31st March 2019.

(3) Depression-candidate Data Set B_D3: To expand the sample size of depressed MSM users, we constructed an unlabeled large data set. Users were identified as depression candidates if their anchor posts loosely contained the character string “depress.” Posts that published within two months before the anchor post of the users were included in this data set.

#### Twitter Database

To get non-MSM samples for comparison, we obtained a publicly available large-scale benchmark Twitter database (18, 37), which is also used in this paper to validate the performance of the XGBoost algorithm. The Twitter database contains three data sets:

(1) Depression Data Set T_D1: Users were labeled as depression if their anchor tweets satisfied a strict pattern “(I’m/I was/I am/I’ve been) diagnosed depression.” Tweets published within one month before the anchor tweet were included in this data set.

(2) Non-Depression Data Set T_D2: Users were labeled as non-depressed if they had never posted any tweet containing the character string “depress.” Tweets published on December 2016 of these users were selected in this data set.

(3) Depression-candidate Data Set T_D3: Based on the tweets in December 2016, users were selected as depression candidates in this unlabeled data set if their anchor tweets loosely contained the character string “depress.”

### Data Description

To further clean the MSM data set, we removed noisy samples from the Blued database for which the number of postings was less than five. Inspired by the work of Shen et al. (18), we also removed accounts that had 15,000 or more followers, which may be organizations or bots. Then we manually checked whether the user was MSM according to his posting content, and removed the samples of non-MSM users. Finally, we obtained 346 depressed MSM users, 8,552 non-depressed MSM users, and 2,627 depression candidates. On the other hand, to construct a non-MSM data set based on the Twitter database, we removed 815 accounts, whose tweets satisfied the strict pattern “(I’m/I was/I am/I’ve been) gay.” Then, we obtained 2,364 depressed non-MSM users, 5,041 non-depressed non-MSM users, and 44,536 depression candidate non-MSM users.

As presented in **Table 1**, the Blued database contains 11,525 users and 237,927 posts, and the Twitter database contains 51,101 users and 41,461,047 tweets. The time range of posts on Blued database is two months, while the time range of tweets on Twitter database is one month. It shows that Twitter users were more active online than Blued users, such that a Twitter user published 798.2 tweets per month but a Blued user only published 10.3 posts per month.

**Table 1:** Description on the Blued and Twitter data sets.

Database	Data set	Number of users	Number of posts	Time range of posts
Blued	B_D1	346	19,457	Jan 2012 to Mar 2019
	B_D2	8,552	155,138	Feb 2019 to Mar 2019
	B_D3	2,627	63,332	Jan 2012 to Mar 2019
	Total	11,525	237,927	Two-month time window
Twitter	T_D1	2,353	480,631	Jan 2009 to Dec 2016
	T_D2	4,990	3,956,077	Dec 2016
	T_D3	43,668	37,024,339	Dec 2016
	Total	51,011	41,461,047	One-month time window

### Data Preprocessing

Before feature extraction, data preprocessing is necessary since there are flexible and variant words in the raw data of social media. To deal with the difficulties in word matching and semantic analysis, we carried out the following data preprocessing procedures on the two data sets:

1. Emoji processing. Emojis are incompatible with many text processing algorithms, so we removed the useless emojis from the emoji library (see **Additional file 1**), and then counted emoticons (emojis with sentiment characteristics) separately.
2. Topics processing. Many posts contain particular topic tags, which are noisy for word matching and text processing. Topic tags are published between two “#” in Blued, e.g. “#love#.” and for Twitter, topic tags are published with one “#” like “#love.” Therefore, we removed all topic tags contained in posts, and then counted the number of topic tags separately.
3. Mention processing. There are many mention marks in the texts of posts, e.g. “@someone”; thus; we removed all such character strings, and counted the times of mentions separately.
4. Stemming. We used the stemming algorithm of the Scikit-learn module in Python to unify word representations. For example, “am/is/are/was/were” should be represented as “be” uniformly.
5. Word segmentation. Unlike English texts, Chinese texts should be segmented into words before text analysis. The word segmentation processing was done using the Jieba module in Python.
6. Filtering stop-words. We removed stop-words from the posts, for example, “a” and “an.” The stop-words in Chinese were filtered using the Jieba module in Python after word segmentation. English stop-words were removed by the Scikit-learn module in Python.

### Feature Extraction

Using references from the psychological and behavioral sciences (18, 25), we finally defined and extracted 19 features based on the Blued and Twitter data sets (see **Table 2**), and classified them into four feature groups to describe multidimensional characteristics of users. As presented in **Table 2**, user-level features were extracted from the Blued users and Twitter users, and post-level features were extracted from the posts on Blued (two-month period) and the tweets on Twitter (one-month period).

**Table 2:** Features used in the XGBoost model.

Level	Group	Feature Name	Definition
Use-level	User Profile Features	followings	The total number of users who followed me.
		followers	The total number of users who I followed.
		listNum	The total number of interested groups that I participated in.
Post-level	Social Interaction Features	favorNum	The average number of times each post was favored by others, *favorNum* = *totalFavorNum*/*postNum*, where *totalFavorNum* is the total number of times my posts was favored.
		mentionNum	The average number of times I was mentioned in other’s posts (e.g. @me), *mentionNum = totalMentionNum/postNum*, where *totalMentionNum* is the total number of times I was mentioned by others.
		repostNum	The average number of times each post was reposted/retweeted by others, *repostNum = totalRepostNum/postNum*, where *totalRepostNum* is the total number of times my posts were reposted by others.
		topicNum	The average number of topic tags contained in each post (e.g. #fun# on Blued or #fun on Twitter), *topicNum = totalTopicNum/postNum*, where *totalTopicNum* is the total number of topics included in my posts.
		postNum	The number of my posts in the data sets.
		timeDist	The average numbers of my posts during 24 h, *timeDist* = [*postNum*_0_, *postNum*_1_,*…*, *postNum*_23_], where , *postNum*_1_ *= totalPostNum*_1_*/postNum* is the proportion of posts published at *i^th^* o’clock.
	Emotion Features	posWordNum	The average number of positive words in each post, *posWordNum = totalPosWordNum/postNum*, where *totalPosWordNum* is the total number of positive words included in my posts.
		negWordNum	The average number of negative words in each post, *negWordNum = totalNegWordNum/postNum*, where *totalNegWordNum* is the total number of negative words included in my posts.
		emoNum	The total number of emoticons in my posts.
		posEmoNum	The average number of positive emoticons contained in my posts, *posEmoNum = totalPosEmoNum/postNum*, where *totalPosEmoNum* is the total number of positive emoticons included in my posts.
		negEmoNum	The average number of negative emoticons contained in my posts, *negEmoNum = totalNegEmoNum/postNum* where *totalNegEmoNum* is the total number of negative emoticons included in my posts.
	Linguistic Features	LDATopicWords	The top 15 LDA topic words extracted from my posts, *LDATopicWords* = [*word*_1_, *word*_2_,*…*, *word*_15_].
		antidepressNum	The average number of antidepressant drug names in each post, *antidepressNum = totalAntidepressNum/postNum*, where *totalAntidepressNum* is the total number of antidepressant drug names mentioned in my posts.
		depressWordNum	The average number of times the character string “depress” appeared in each post (named depressive word), *depressWordNum = totalDepressWordNum/postNum*, where *totalDepressWordNum* is the total number of depressive words in my posts.
		picNum	The average number of pictures in each post, *picNum = totalPicNum/postNum*, where *totalPicNum* is the total number of pictures in my posts.
		videoNum	The average number of videos in each post, *videoNum = totalVideoNum/postNum*, where *totalVideoNum* is the total number of videos in my posts.

#### User Profile Features

In this paper, three different user profile features are defined: the numbers of *followings* and *followers* describe the online egocentric social networks of users during their accounts’ lifetime; and *listNum* is the number of interested groups/lists that the user participated in.

#### Social Interaction Features

Based on the number of posts (*postNum*), we obtained the number of times for which each post was favored (*favorNum*) and reposted/retweeted (*repostNum*) by other users, the number of topics mentioned in each post (*topicNum*), and the number of times a user was mentioned in others’ posts (*mentionNum*), to describe the interaction behaviors among users. In addition, the time distribution of postings (*timeDist*) was collected to characterize the active time of users on social networks.

#### Emotion Features

There are many differences in emotional status between depressed users and non-depressed users; thus, emotional features are beneficial for depressive emotion detection. We counted the numbers of positive words (*posWordNum*) and negative words (*negWordNum*) in each post. These positive and negative words in English and in Chinese are extracted by the Scikit-learn module in Python and dictionary-based methods, respectively. The number of emoticons (*emoNum*) from the posting content was counted as well as the numbers of positive emoticons (*posEmoNum*) and negative emoticons (*negEmoNum*). Six volunteers voted on the sentiment (positive, negative, and neutral) of the emoticons, as shown in **Additional file 1**.

#### Linguistic Features

With respect to the content characteristics of users’ postings, firstly, the topics expressed by depressed users and non-depressed users are likely to differ significantly. Therefore, we applied the unsupervised latent Dirichlet allocation (LDA) model to extract 15 salient LDA topic words (*LDATopicWords*) from users’ posts. Second, there are some domain-specific features from depressed users, such as the number of antidepressant drug names (*antidepressNum*) and the number of depressive words which containing the character string “depress” (*depressWordNum*). The names of the antidepressants in Chinese and in English are presented in **Additional file 2**. Finally, the media types of posts were also collected, such as the numbers of pictures (*picNum*) and videos (*videoNum*).

### Classification Method

Let *D* ={(*x_i_*, *y_i_*)}(|D| = *n*, *x_i_* ϵ *R^n^*,*y_i_* ϵ *R^n^*) represents a data set with *n* examples and *m* features. The classification method aims to find a relationship between an input *X* = {*x*_1_, *x*_2_,…, *x_N_*,} and an output *Y*. In our case, we determined whether a user was depressed based on four feature groups, and we aimed to determine the best combinations of features that would show the most predictive power in this binary classification. We used the XGBoost (eXtreme Gradient Boosting) (38) algorithm for this purpose, which is a well-designed gradient-boosted decision tree (GBDT) (39) algorithm that has demonstrated its state-of-the-art advantages in scientific research for machine learning and data mining. XGBoost belongs to a group of widely used tree learning algorithms, which do not require linear features or linear interactions between features. A decision tree allows for making predictions on an output variable based on a series of rules arranged in a tree-like structure. In this paper, all users (samples) were described by the set of 19 features that were classified into four groups. The XGBoost algorithm was implemented on the training data sets (D1 and D2) using the Scikit-learn Python (40) libraries for machine learning processes. To prevent overfitting and to make a good assessment of model validity, we employed a stratified five-fold cross validation to conduct the experiments with 12 randomized experimental runs in order to reduce variance.

#### Experiment Setting

The optimal values of parameters for the XGBoost algorithm were carefully tuned by a grid search with small but adaptive step size to enumerate the classification accuracy rates in different XGBoost parameter settings. We choose six estimator values of 50, 100, 200, 300, 400, and 500, five minimum child weight values of 1, 2, 3, 4, and 5, and five regularization alpha values of 1e-5, 1e-3, 1e-2, 0.1, and 1. The search ranges for learning rate, maximum tree depth, subsample and colsample_bytree were [0.05,0.2], (3, 11), [0.5,1] and [0.5,1], respectively. We carefully tuned the parameters of the XGBoost algorithm to obtain the best performance, the final XGBoost model parameter settings are described in **Table 3**.

**Table 3:** Parameters of XGBoost model for the Blued and Twitter data sets.

	Parameter	Blued	Twitter
XGBoost	Estimators	50	200
	Maximum tree depth	4	4
	Learning rate	0.06	0.06
	Minimum child weight	2	1
	Subsample	0.45	0.8
	Colsample_bytree	0.65	0.8
	Regularization alpha	1	0.001

#### Metrics

We evaluate the detection performance of XGBoost using four widely used metrics, i.e. accuracy, macro-averaged recall (Recall), macro-averaged precision (Precision), and macro-averaged F1 score (F1 score). For the classification task in this paper, the terms *true depressed*, *true non-depressed*, *false depressed* and *false non-depressed* compare the results of the classifier.

These four metrics can be counted by the following formulas:

$$Accuracy=\frac{\Sigma \;true\;depressed+\Sigma \;true\;non\_depressed}{\Sigma \;total\;population}$$

$$Recall=\frac{\Sigma \;true\;depressed}{\Sigma \;true\;depressed+\Sigma \;false\;non\_depressed}$$

$$Precision=\frac{\Sigma \;true\;depressed}{\Sigma \;true\;depressed+\Sigma \;false\;depressed}$$

$$F1score=2\cdot \frac{Precision\cdot Recall}{Precision+Recall}$$

## Results

We first validated the effectiveness of the XGBoost model on the labeled Blued data sets (B_D1 and B_D2) and Twitter data sets (T_D1 and T_D2) and compared the importance of each extracted feature for the model. Next, we applied the trained XGBoost model to the unlabeled depression-candidate data sets (B_D3 and T_D3), in order to obtain a large number of depressed users. Finally, based on the massive social networking data of MSM and non-MSM users, we analyzed the differences in online behaviors between depressed MSM and non-depressed MSM, as well as the differences between depressed MSM and depressed non-MSM.

### Model Performance

The classification accuracy rates on the Blued and Twitter data sets are displayed in **Table 4**. It can be seen that the XGBoost achieved outstanding performance on both the Blued data sets and the Twitter data sets. Given that certain online behavior characteristics and patterns of users can be found on online social networks, similar characteristics might be found on different kinds of social networks. The high performance on both the Blued data set and the Twitter data set also indicates that depressive emotion detection methods *via* online behavioral feature learning techniques possess strong applicability and generality for different social networking platforms. Thus, other sources of online social networks, such as Facebook and Instagram, would also provide multidimensional online information for depressive emotion detection.

**Table 4:** Classification performance of the XGBoost algorithm on the Blued and Twitter data sets.

Data set	Accuracy	Recall	Precision	F1 score
Blued	0.9940	0.9648	0.9563	0.9602
Twitter	0.9671	0.9591	0.9649	0.9619

**Figure 1:**
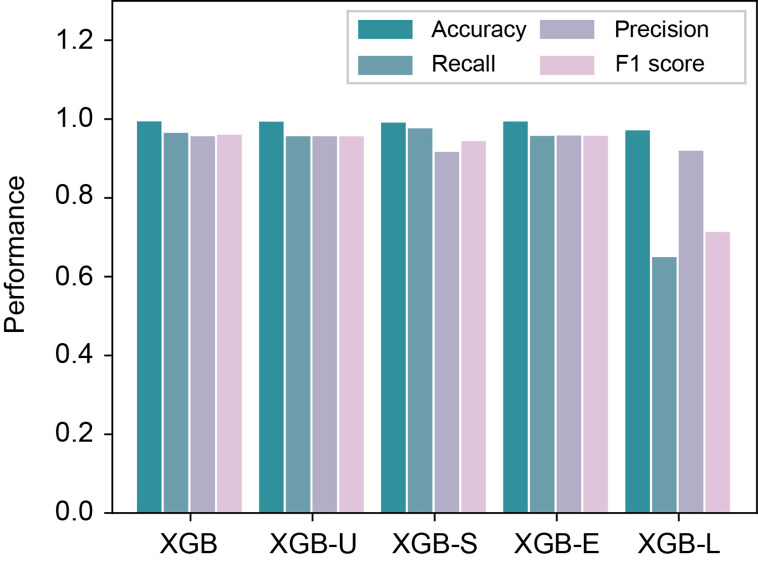
Performance comparison of different feature combinations in the Blued data sets.

In order to study the effectiveness of different feature combinations, we then constructed an experiment to feed our model with one feature group removed each time based on the Blued data sets. Specifically, we first used all feature groups, denoted as XGB. We then removed the four feature groups separately and denoted them as XGB-U (for removing user profile features), XGB-S (for removing social interaction features), XGB-E (for removing emotion features) and XGB-L (for removing linguistic features), respectively. From the results shown in **Figure 1**, we can see that the XGB-L performed worse than the others, indicating that linguistic features are more significant than the other feature groups. Furthermore, the social interaction features also contribute much to the performance, which shows that depressed users usually have different social networking behaviors.

### Feature Importance

**Figure 2:**
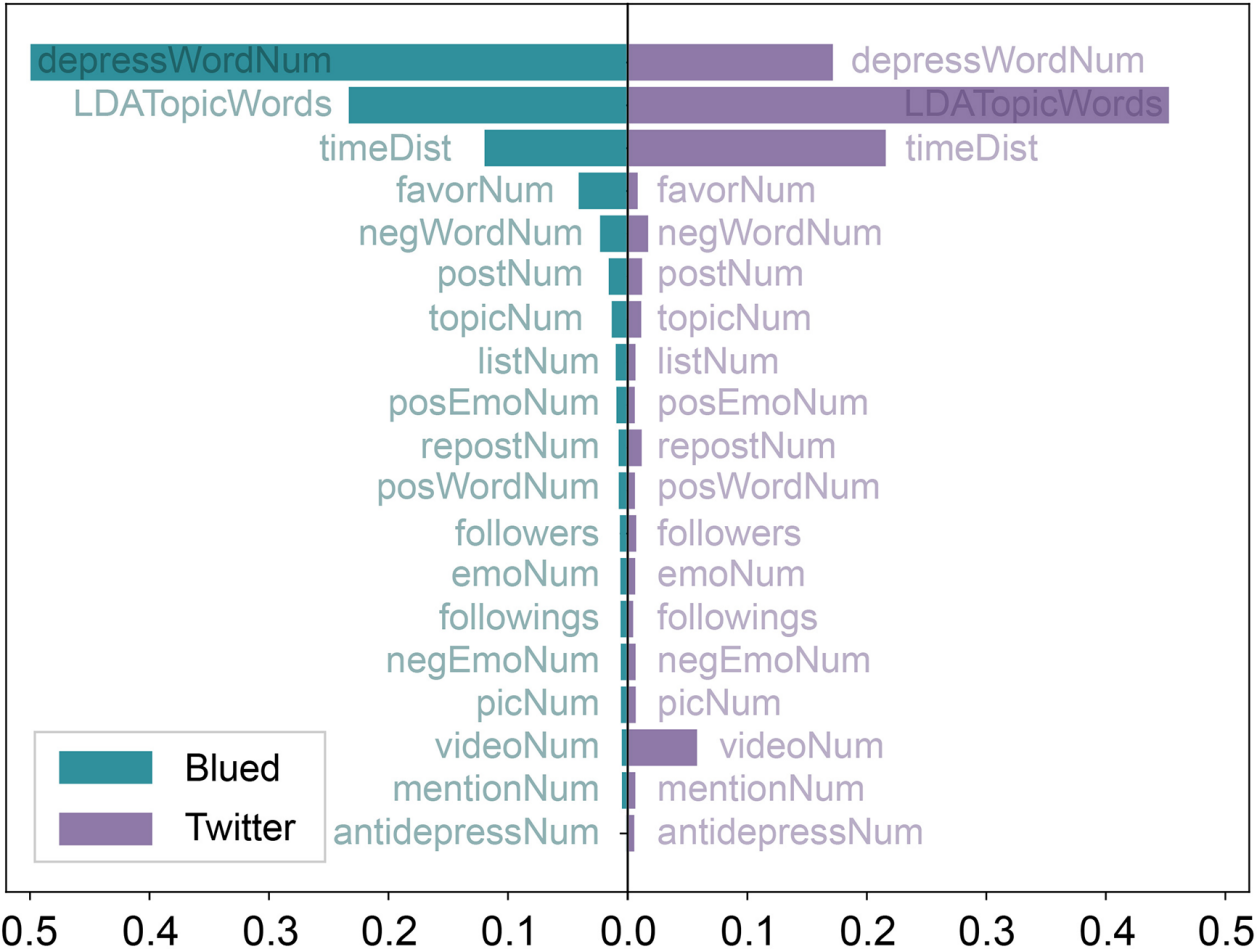
Feature importance of XGBoost algorithm in the Blued and Twitter data sets.

Feature importance gives a score for each feature of our data, and the higher the score, the more important or relevant the feature is towards the output (depressive emotion detection result). In this paper, the feature importance of each feature in the XGBoost algorithm is a fraction of the number of times the feature was used to split the data across all decision trees among the number of times all features are used to split the data across all decision trees. **Figure 2** shows the importance of each feature in the XGBoost model for classification on the Blued data sets (B_D1 and B_D2) and Twitter data sets (T_D1 and T_D2). The three most important features in both Blued and Twitter data sets are *depressWordNum*, *LDATopicWords* and *timeDist*, which held about 84% of feature importance in total. It suggests that these three features are more significant than other features in reflecting the different online behaviors of depressed users and, thus, contribute most to depressive emotion detection.

**Figure 3:**
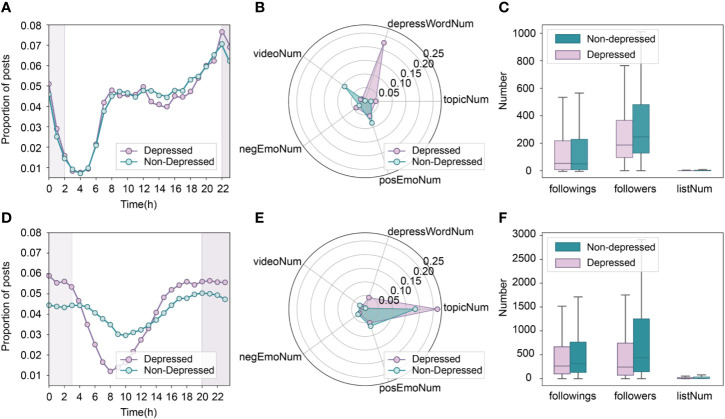
Online behavior characteristics of depressed and non-depressed users for MSM population on Blued (top row) and non-MSM population on Twitter (bottom row). **(A**, **D)**: active time comparison; **(B**, **E)**: posting custom of each post with regard to five representative behaviors; **(C**, **F)**: distributions of user profile features.

**Figure 4:**
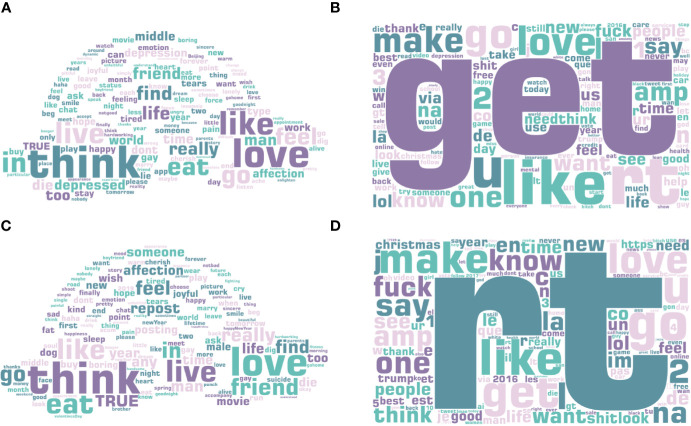
Salient LDA topic words for **(A)** depressed MSM users, **(B)** depressed non-MSM users, **(C)** non-depressed MSM users, and **(D)** non-depressed non-MSM users. The larger the word, the more frequent it appears in the posts.

There are a number of differences in feature importance between Blued and Twitter data sets. The most crucial feature in Blued was *depressWordNum*, which comprised nearly half of feature importance, however, in Twitter, *depressWordNum* only held less than 20% of feature importance. There is an obvious difference in the usage of depressive word between depressed and non-depressed Blued users, such that each depressed Blued user in B_D1 has published at least one post that contained the character string “depress” and 99.2% of non-depressed Blued users in B_D2 have never published depressive words. Different from the depressed Blued users, 7.18% of depressed Twitter users in T_D1 have never posted depressive words and 81.4% of non-depressed Twitter users in T_D2 have never published depressive words. Affected by the outstanding classification power of *depressWordNum*, the *LDATopicWords* became the second crucial feature in Blued but held about a half of feature importance in Twitter, though the difference between depressed and non-depressed users are similar in Blued and Twitter data sets (see **Figure 4**). The feature importance of *timeDist* is larger in Twitter than in Blued, which can be explained by the more obvious differences observed between depressed and non-depressed users in Twitter, as shown in **Figures 3A, D**.

An interesting finding is that *videoNum* was the fourth important feature in Twitter data sets, but was an unimportant feature in Blued data sets. However, we find that the difference in *videoNum* between depressed and non-depressed users is more obvious in Blued data sets (mean diff = 0.07) than in Twitter data sets (mean diff = 0.02). One reason for the inconsistency might be the relatively lower feature importance of *videoNum* (5.7%) compared with the three most crucial features (83.7%). That is, *videoNum* is the fourth important feature in Twitter, however, compared with the difference between depressed and non-depressed users in *LDATopicwords*, *depressWordNum* and *timeDist*, the difference in *videoNum* become less obvious. In general, the results of feature importance in Blued and Twitter data sets indicate that on the one hand, the dominating explainable features share many similarities, i.e., both data sets have *depressWordNum*, *LDATopicWords* and *timeDist* as the most important features; on the other hand, there are considerable differences between MSM and non-MSM users regarding the order of the most crucial features and the *videoNum* feature.

### Behavior Analysis

All users on both Blued (MSM users) and Twitter (non-MSM users) can be classified into four user groups: depressed MSM, non-depressed MSM, depressed non-MSM, and non-depressed non-MSM. After applying the XGBoost algorithm to the depression-candidate data sets B_D3 and T_D3, we detected 1,445 depressed MSM users and 24,748 depressed non-MSM users. Then, the numbers of depressed MSM users and non-depressed MSM users are 1,791 and 9,734, respectively. And the numbers of depressed non-MSM users and non-depressed non-MSM users are 27,101 and 23,910, respectively. We analyzed the difference of online behaviors between different categories of users, the mean results of the extracted features are presented in **Table 5**, **Figures 3** and **4**.

**Table 5:** Mean of features on Blued data sets and Twitter data sets.

Feature	MSM users (Blued)			Non-MSM users (Twitter)		
	Depressed	Non-depressed	Total	Depressed	Non-depressed	Total
followings	254.491	306.236	298.2 (0~29,798)	676.427	748.8731	710.384 (0~16,890)
followers	514.511	550.949	545.3 (0~82,409)	888.671	1237.16	1052.01 (0~14,994)
listNum	1.9	2.758	2.6 (0~20)	35.313	52.8384	43.53 (0~9,313)
postNum	28.675	19.167	20.6 (1~1,826)	392.887	1214.16	777.834 (1~3,260)
repostNum	0.0227	0.0798	0.07 (0~57.19)	1201.23	1558.16	1368.53 (0~422,814)
mentionNum	0.0004	0.0028	0.002 (0~2.19)	0.605	0.6466	0.624 (0~8.636)
topicNum	0.0394	0.022	0.025 (0~6.8)	0.265	0.184	0.227 (0~10.535)
picNum	0.966	1.371	1.31 (0~9)	0.17	0.21	0.189 (0~3.06)
videoNum	0.0129	0.0921	0.08 (0~1)	0.0155	0.024	0.019 (0~1)
antidepressNum	0.0003	3.453	0.00007 (0~0.25)	0.013	0.0122	0.0126 (0~1.01)
depressWordNum	0.225	0.0019	0.037 (0~4)	0.0447	0.0039	0.025 (0~2.988)
emoNum	0.098	0.112	0.109 (0~2.5)	0.0916	0.112	0.101 (0~3.286)
posEmoNum	0.057	0.084	0.079 (0~2)	0.0533	0.0655	0.059 (0~1.971)
negEmoNum	0.039	0.026	0.028 (0~1.5)	0.026	0.0325	0.029 (0~2.5)
posWordNum	2.368	1.41	1.559 (0~33)	0.434	0.339	0.39 (0~6)
negWordNum	2.079	0.992	1.161 (0~24)	0.339	0.266	0.305 (0~6)
favorNum	7.618	23.622	21.135 (0~1353.73)	1.0065	0.976	0.992 (0~597.875)

#### Comparison Between Blued Users and Twitter Users

There are many differences in online behaviors between Blued users and Twitter users, since the purposes of users in Blued and Twitter may show some differences to some extents. As shown in **Table 5**, with an enormous number of active users, Twitter users had more followings and followers, and attended more interested groups. Regarding the posting behaviors, Twitter users posted more tweets than Blued users, and each tweet contained a larger number of topics, pictures, videos, antidepressant drug names, and depressive words. In comparison with Twitter users, Blued users posted more emoticons, positive emoticons, positive words, and negative words. In addition, while tweets on Twitter are more likely to be retweeted than the posts on Blued caused by the purpose of information sharing, posts on Blued are more likely to be favored by others, driven by the purpose of seeking partners. Concerning LDA topics, we compared the top 30 salient LDA topic words between Blued and Twitter data sets, and found that 30% of the words were similar. These similar words were commonly used in daily life, such as “feel,” “go,” “life,” “like,” “love,” “really,” “repost,” “time,” and “year.”

The difference between Blued users and Twitter users, to a certain extent, reveals that MSM and non-MSM users show different online social behaviors. For example, the active online interaction among Twitter users might indicate that non-MSM users are more active online than MSM users and have more diversified forms to express their feeling and emotion, such as pictures and videos. In addition, MSM users prefer directly expressing their emotions and feelings, thus their posts contained more emoticons and emotion words.

#### Behavior Analysis of Depressed and Non-Depressed MSM

First, we examined the users’ posting proportions at different time periods, as shown in **Figure 3A**. It was found that MSM users tend to publish more posts between 6 pm and 1 am, indicating that the MSM population prefers frequently using social networks at night. Another possible explanation is that they are likely to suffer from insomnia. It also appears that the degree of going to bed late or suffering from insomnia is higher among depressed MSM than non-depressed MSM, since depressed MSM published more posts between 10 pm and 2 am.

Next, the user posting patterns and user profile features were analyzed. As shown in **Figure 3B**, depressed MSM users posted on average 0.225 depressive words and 0.039 negative emoticons per post, which surpassed the non-depressed MSM users by 0.223 and 0.013, respectively. This suggests that depressed MSM users may express their depressive emotions and complain more about their bad moods. In addition, the depressed MSM users published more posts that containing more topics but fewer videos than non-depressed MSM users. With regard to *negWordNum* and *posWordNum*, as presented in **Table 5**, we found that depressed MSM users posted more negative words (1.087) and positive words (0.958) than non-depressed MSM users. From **Figure 3C**, we can see that the differences between the average numbers for the followings and interest groups are small between depressed MSM and non-depressed MSM. In addition, the non-depressed MSM has more followers than the depressed MSM, indicating that the non-depressed MSM users are more likely to be favored or followed by others.

Finally, we constructed the word cloud of the top 150 salient terms of LDA topics, as shown in **Figures 4A, C**. The content of the posts was so casual that both the depressed MSM users and non-depressed MSM users published posts containing many commonly used words, such as “love,” “like,” “live.” In addition, their posts included words such as “eat,” “go,” “life,” and “friend,” indicating that both classes share their daily lives on Blued. However, depressed MSM users are more likely to be under pressure, as their posts included more occurrences of “work” and “don’t,” while the posts of non-depressed MSM users contained more words like “movie” and “sleep.” In addition, in contrast to the posts of non-depressed MSM users, the posts of depressed MSM users involved words such as “depressed,” “depression,” and “die,” which implies that depressed MSM users tend to post their mental status on Blued and they have significant mental health problems.

For the remaining features, in comparison with the depressed MSM users, we discovered that the non-depressed MSM users’ posts are more likely to be favored and reposted by others. Furthermore, the non-depressed MSM users publish more posts containing more pictures, and they also prefer mentioning other users in their posts.

#### Behavior Analysis of Depressed MSM and Non-MSM Users

As shown in **Figures 3** and **4**, the non-MSM users show a clear posting pattern of being active at nighttime and silence during the daytime. Compared to non-MSM users, MSM users maintain a high level of activity most of the time except in the small hours. This shows that MSM users almost cannot survive without online social networks, and they have a stronger virtual-social-dependence than non-MSM users.

Similar to depressed MSM users, the depressed non-MSM users posted more depressive words than non-depressed non-MSM users, and the content of their posts contained more topic tags. An obvious difference is that the posting content of depressed MSM users contained 0.18 more depressive words than that of depressed non-MSM users, indicating that depressed MSM users complain more and they are more likely to express their negative emotions on social networks. In addition, depressed non-MSM users published posts with 0.226 more topic tags than depressed MSM users, which is related to the differences in the target users and design principles between Twitter and Blued. The former has a-broad-audience and a stronger information dissemination capacity. Similarly, non-MSM users have more followers and followings than MSM users, since Twitter has more than 500 million users while Blued only has about 40 million users.

An interesting finding is that depressed MSM users posted 2.079 negative words and 2.368 positive words, surpassing that of depressed non-MSM users by 1.74 and 1.934, respectively (see **Table 5**). The same results can be seen between non-depressed MSM users and non-depressed non-MSM users. This again shows that the MSM population prefers to express their emotions on social networks compared to the non-MSM population. Another interesting finding is that non-depressed non-MSM users posted more negative emoticons than depressed non-MSM users, which differs from that of the MSM users. In comparison with depressed MSM users, depressed non-MSM users published posts with more positive LDA topic words, such as “good,” “new,” “thank,” and less negative LDA topic words, such as “depression,” “depressed,” and “dont,” as shown in **Figures 4A, B**.

## Discussion

Overall, we aimed to investigate the feasibility of automated detection of depressive emotions among the MSM population using social networking data on online mass media. With the well-labeled data sets and well-defined depression-oriented features, the XGBoost algorithm achieved good performance on detecting depressed users for both MSM users on Blued and non-MSM users on Twitter. We further analyzed the contribution of the feature groups and the importance of each feature in the XGBoost algorithm. In addition, based on the depressive emotion detection results, we analyzed the differences between Blued users and Twitter users, the differences between depressed and non-depressed MSM users, and the differences between depressed MSM and non-MSM users. Compared to non-MSM users, MSM users complain more and are more likely to express their negative emotions on social networks.

In our study, we adopted a machine learning model named XGBoost to implement depressive emotion detection. In comparison with deep learning models which provide powerful predictive capability but generally lack interpretability, the XGBoost model is a kind of decision tree-based ensemble method which can provide interpretability to some extent in feature extraction and decision processes as well as achieve outstanding performance in data mining fields and classification tasks. Since our work focused on the depressive emotion detection and behavior analysis of MSM population, the XGBoost algorithm is capable of achieving these goals. And based on the results achieved by the XGBoost model, we can further analyze the importance of each feature in the depressive emotion detection task.

The linguistic feature group is more significant than the other feature groups, when using XGBoost algorithm to detect depressive emotions. The linguistic features include LDA topic words, depressive words, the number of pictures, and the number of videos in the posts, which reflect the content characteristics of the users’ online posts. Similar with the linguistic features in reality, the online linguistic feature is also a potential reference for identifying the mental health of users (41). Additionally, in the depressive emotion detection task, the most important features for determining the detection accuracy are depressive words, LDA topic words, and posting time distribution. These features are useful for describing depressive symptoms, such as depressed mood, users’ feelings, social behaviors, and sleep disturbance, which are also used as diagnosing indicators in many scales to assess depression (17). Therefore, the XGBoost model can extract multi-dimensional depressive symptoms of users through massive online social networking data, and then help detect depression in the early stage.

In addition, while most studies conducted by questionnaire and interview were limited by the sample size of participants and multidimensional characteristics of social behaviors, our work suggests that the automated depressive emotion detection using online mass media data with a machine learning manner, is a potential and easy-to-use way to achieve both large-scale samples and multidimensional depressive symptoms detection. Evidence has shown that many depressive symptoms that are used for clinical depression diagnoses are reflected in online social behaviors, such as insomnia, suicidal thoughts, and depressive mood (21, 29, 41). Thus, further diagnosis or medication of depression is needed for depressed users, as they are found to have significant mental health problems.

Regarding MSM population who are at high risk of HIV and experience remarkably poorer mental health, few studies have focused on the depressive emotion detection of MSM, and the differences between depressed and non-depressed MSM, as well as between MSM population and non-MSM population. In our work, we provide an innovative approach for the monitoring of depressive emotions or mental health among MSM population *via* social media. It is of significant importance in this at-risk population as it has been reported that depression is associated with increased risk for HIV in MSM population. The digital monitoring of depressive symptoms of MSM population in this paper can complement and extend traditional approaches to diagnose depression, and enable those suffering depressive emotions to be more proactive about their mental health. Additionally, compared to survey methods to recruit MSM populations with depression, we expect our findings to provide more perspectives and insights for MSM-related researches in online data collection and big data analysis.

However, in our study, depressed MSM users were identified if they published posts saying they are depressed and have suicidal thoughts; this kind of depressive emotion identification method is poor and inaccurate compared to the clinical depression diagnoses and the depression diagnosis measure in the benchmark Twitter data set. Furthermore, the non-MSM data set was constructed from the Twitter data set by removing the accounts of those who published posts describing themselves as gay; this kind of identification measure is also weak to a certain extent. Fortunately, based on the well-labeled Twitter data set, the rationality of the algorithm was proved with a good detection performance.

## Conclusion

In summary, this is a new attempt to detect depressive emotions among MSM population using massive online social networking data with a machine learning algorithm. An effective and easy-to-use method is provided here for monitoring depressive emotions, which can help identify at-risk individuals in the early stage of depression for further clinical diagnosis. In addition, this is a novel analysis of the differences between MSM population and non-MSM population with or without depressive emotions. Automated depressive emotion screening *via* social media is a feasible and efficient measure for both the general population and hard-to-access populations. In the future, we expect to improve the representativeness of MSM population samples from online social media data and research the association between depression and stigma, and the sexual risk behaviors in MSM with or without HIV *via* online recruitment methods.

## Data Availability Statement

The Twitter data used in this article are available at http://depressiondetection.droppages.com and the Blued data can be accessed under reasonable requests from the authors.

## Ethics Statement

Ethical review and approval was not required for the study on human participants in accordance with the local legislation and institutional requirements.

## Author Contributions

XL and MC designed the research. MC and YL performed the experiments and wrote the paper. SQ designed all the data visualizations. YL, XL, and SQ helped to revise the manuscript. All authors contributed to the article and approved the submitted version.

## Funding

XL acknowledges the Natural Science Foundation of China (91846301, 71771213, and 71790615) and the Hunan Science and Technology Plan Project (2017RS3040, 2018JJ1034). YL is supported by the Natural Science Foundation of Hunan Province (2019JJ40328, 2019GK2131). MC is supported by the Natural Science Foundation of China (71690233, 71774168) and China Scholarship Council (CSC201903170182). The funding bodies had no role in the study design, data collection, analysis, interpretation of the data, and in writing the manuscript. The opinions expressed here represent those of the authors and do not necessarily reflect the views of the funders.

## Conflict of Interest

The authors declare that the research was conducted in the absence of any commercial or financial relationships that could be construed as a potential conflict of interest.

## Supplementary Material

Additional file 1 emoji_and_emoticon_library.xlsx.

Additional file 2 antidepressant_table.xlsx.
